# Geographic distribution of suitable hosts explains the evolution of specialized gentes in the European cuckoo *Cuculus canorus*

**DOI:** 10.1186/1471-2148-9-88

**Published:** 2009-04-30

**Authors:** Juan J Soler, Manuel Martín Vivaldi, Anders Pape Møller

**Affiliations:** 1 Departamento de Ecología Funcional y Evolutiva, Estación Experimental de Zonas Áridas (CSIC), General Segura 1, E-04001 Almería, Spain; 2 Departamento de Biología Animal, Universidad de Granada, E-18071 Granada, Spain; 3 Laboratoire d'Ecologie, Systématique et Evolution, CNRS UMR 8079, Université Paris-Sud, Bâtiment 362, F-91405 Orsay Cedex, France

## Abstract

**Background:**

Several types of selective forces can act to promote parasite specialization. Parasites might specialize on some suitable hosts at the cost of decreasing effectiveness when exploiting other species of hosts, and specialization can be more easily selected for in hosts that the parasites will easily find. Thus demographic characteristics of suitable hosts such as population density and its spatial consistency could be key factors predicting probability of parasite specialization and speciation. Here, we explore this hypothesis by studying the relationship between occurence of specialized races of the European cuckoo (*Cuculus canorus*) (i.e. gentes) and mean and coefficient of variation in population density estimated for 12 different European regions.

**Results:**

The results were in accordance with the hypothesis because specialized cuckoo egg morphs were more common in suitable hosts with high population density and low variation in population density at the level of host species or genera.

**Conclusion:**

We have presented evidence suggesting that population density and homogeneity of geographic distribution of hosts explain, at least partly, the evolution of specialized egg-morphs of the European cuckoo. These results are consistent with the hypothesis that resource (i.e., host) predictability explains the evolution of host races and species of parasites.

## Background

The study of evolutionary processes of host specialization by parasites is of special importance for evolutionary biologists because it causes speciation events in parasites, and, therefore, it is one of the main examples of ecological conditions driving adaptive radiation of parasitic groups on their hosts [1,2]. In general, among suitable hosts, the degree of host specificity of parasites can be seen as an equilibrium between two opposing evolutionary forces that describe the trade-off in the ability to optimally parasitize different hosts: (1) to use the maximum number of hosts encountered; and (2) to make best use of the most frequently encountered hosts [3]. However, as the number of potential host species increases, the probability that a specialist parasite can locate a suitable host decreases, reducing the advantages gained through specialization. In these cases, generalist parasites would experience greater fitness advantages and specialization would no longer be adaptive [4]. Thus, when trade-offs for exploiting different hosts exist, relative host abundance is the key to host specificity [3]. Given sufficient abundance of target host, the benefits of specialization, which increase the parasite's efficiency in acquiring resources from the most abundant host, outweigh the disadvantages of interacting less well with other non-specific potential hosts [4]. On the other hand, generalist parasites would thrive in heterogeneous communities, as it allows a parasite to reproduce successfully in many of the encountered potential hosts. In other words, predictability of resources (i.e., hosts availability) would be a key factor explaining probability of host specialization [5]. If host populations are unpredictable and ephemeral, generalist parasites are more likely to occur [6].

Demographic parameters of both host and parasites including host abundance and distribution at a large geographic scale (i.e. metapopulation level) are known to be key factors explaining host-specialization by parasites [7]. Many parasites show common patterns of host specificity, with higher host specificity where host abundance is high and reliable [3,7]. In accordance with this pattern, in several unrelated parasitic taxa, greater host specificity has been detected in temperate, but not in tropical, regions where the community of potential hosts is less diverse and species are more abundant [see examples in [3]]. In addition, in a study involving five trophic levels (plant, phytophage, parasite, parasitoid and hyper-parasite), a high degree of specialization was detected at the lower trophic levels correlating with a greater relative abundance of host species [8]. Thus, demographic characteristics (relative abundance and distribution) of potential hosts were in fact shown to predict the evolution of specialized races or species of parasites [3].

Here we explore this hypothesis in the European cuckoo, a generalist brood parasite with host races specializing in parasitizing particular host species [9-11]. As any interspecific brood parasite, the European cuckoo lays its eggs in the nests of other bird species, the hosts that incubate and raise parasitic offspring. Because cuckoos often impose severe fitness costs on parasitized hosts, recognition and rejection of parasite eggs is of selective advantage for hosts. On the other hand, mimicry of parasitic egg to those of their hosts is advantageous for cuckoos because it reduces the probability of hosts recognizing and rejecting parasitic eggs. However, because egg appearance of different cuckoo host species may drastically differ, and a cuckoo female lay eggs of a specific colour pattern [12], but cannot change the appearance of their eggs depending on the parasitized host species, the advantage of egg mimicry when parasitizing hosts with similar eggs became disadvantageous when parasitizing other suitable hosts. Consequently, the evolution of different cuckoo egg morphs that mimic eggs of their hosts reflects specialization occurring in some, but not all suitable host species [13,14]. In accordance with the hypothesis of demographic characteristics of hosts affecting the resolution of the trade-off for the ability to adapt optimally to different hosts (see above), we predicted that specific egg-morphs of European cuckoos should have evolved in hosts that are evenly distributed at high density in their European range.

**Figure 1:**
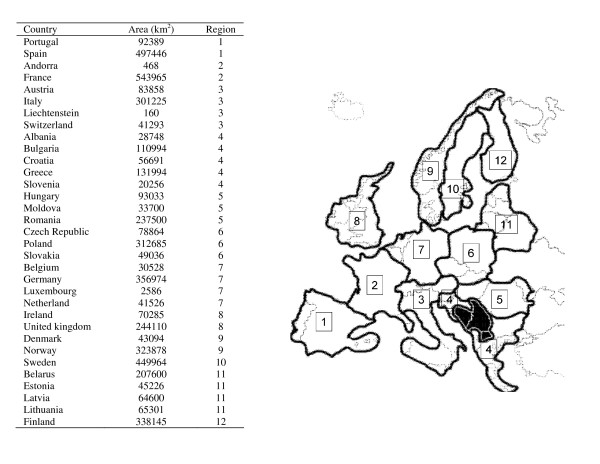
**European countries within the twelve regions considered in the analyses**. Information on areas (km^2^) of different countries is also provided.

To test this prediction suitable host species or genera were classified as having or not having specialized egg morphs (i.e., specific cuckoo gentes) following Moksnes and Røskaft [9]. We collected information on population density of suitable host species for 12 European geographic regions of similar area (Fig. 1). As an index of heterogeneity in population density, we estimated the coefficient of variation (CV) in density among these twelve European regions as an index of spatial distribution widely used in ecology for characterizing uniformly (CV < 1), randomly (CV = 1) or contagiously (CV > 1) distributed species [e.g., [15]].

## Results

As expected from the hypothesis of parasite specialization being more common in abundant and evenly distributed hosts, we found that European cuckoos have evolved host-specific egg morphs for host species with higher population density and lower coefficients of variation in population density in different regions. That was the case independent of whether the analyses were based on species-specific data or phylogenetically independent contrasts (Table 1). These relationships were independent of the allometric effects of body mass because the inclusion of phylogenetic independent contrasts of body mass in a multiple regression through the origin did not explain the probability of evolution of specialized cuckoo-egg morphs (with population density as a second independent variable: Beta (SE) = 0.154 (0.109), t_62 _= 1.41, P > 0.15); with CV of population density as a second independent variable: Beta (SE) = 0.174 (0.109), t_62 _= 1.60, P > 0.1), and it did not affect the percentage of variance explained by population density (Beta (SE) = 0.260 (0.109), t_62 _= 2.52, P = 0.02) or by coefficient of variation in population density (Beta (SE) = -0.287 (0.109), t_62 _= 2.64, P = 0.01). Furthermore, the addition of duration of the nestling period (the second variable explaining host selection by cuckoos [16] to the model did not affect the conclusions (Table 2)

**Table 1:** Among species and genera comparisons (I).

		With gentes			Without gentes	Statistical tests for log-transformed variables
		N	Mean (SE)	N	Mean (SE)	t-tests
		
***Analyses with*** ***species information***	Log population density	10	-0.265 (0.444)	69	-2.078 (0.324)	t = 2.08, P = 0.041
	Log CV of population density	10	4.764 (0.190)	69	5.274 (0.059)	t = 3.00, P = 0.004
	
	*Phylogenetically independent contrasts*		Beta (SE)		Corrected df	Regression through the origin
		
	Log population density		0.258 (0.110)		63	t = 2.35, P = 0.022
	Log CV of population density		-0.272 (0.110)		63	t = 2.49, P = 0.016

***Analyses with*** ***genus information***		N	Mean (SE)	N	Mean (SE)	t-tests
		
	Log population density	10	1.304 (0.308)	21	-0.960 (0.474)	t = 3.13, P = 0.004
	Log CV of population density	10	4.353 (0.151)	21	5.160 (0.111)	t = 4.22, P = 0.001
	
	*Phylogenetically independent contrasts*		Beta (SE)		Corrected df	Regression through the origin
	
	Log population density		0.464 (0.164)		27	t = 2.82, P = 0.015
	
	Log CV of population density		-0.580 (0.151)		27	t = 3.83, P = 0.001

Among species and genera comparisons of mean and coefficient of variation (CV) in population density of suitable host passerine species for which the European cuckoo has or has not evolved specific gentes (i.e., highly mimetic egg morphs). Possible phylogenetic dependence of data was taken into account by using pair-wise comparisons of closely related taxa (i.e., mean values of species with and without gentes within families) and phylogenetically independent contrasts.

**Table 2:** Between species comparisons (II).

*Raw data: GLZ (Binomial distribution and logit link function). Dependent variable: with vs without specialized cuckoo egg morphs*				
	Population Density	CV population density	Body mass	Duration of nestling period

Model 1	χ^2 ^= 5.17, P = 0.023		χ^2 ^= 0.74, P = 0.39	χ^2 ^= 0.66, P = 0.42
Model 2		χ^2 ^= 7.72, P = 0.005	χ^2 ^= 0.86, P = 0.35	χ^2 ^= 0.65, P = 0.42

*Phylogenetically independent contrasts: Multiple regression throughout the origin*				

	Beta(SE)	Beta(SE)	Beta(SE)	Beta(SE)
Model 1	0.24(0.11), P < 0.04		0.16(0.11), P > 0.1	0.09(0.11), P > 0.4
Model 2		-0.28(0.11), P = 0.01	0.19(0.11), P > 0.1	0.12(0.11), P > 0.2

Comparisons between species for which the European cuckoo has and has not evolved specialized egg morphs. Raw data were compared by means of Generalized Linear Models and Chi-square statistic (df = 1 for all performed analyses). Phylogentically independent contrasts were compared by means of regression analyses through the origin (corrected df = 64 for all performed analyses). Model 1 and model 2 respectively include population density and coefficient of variation (CV) in population density as independent variables.

**Figure 2:**
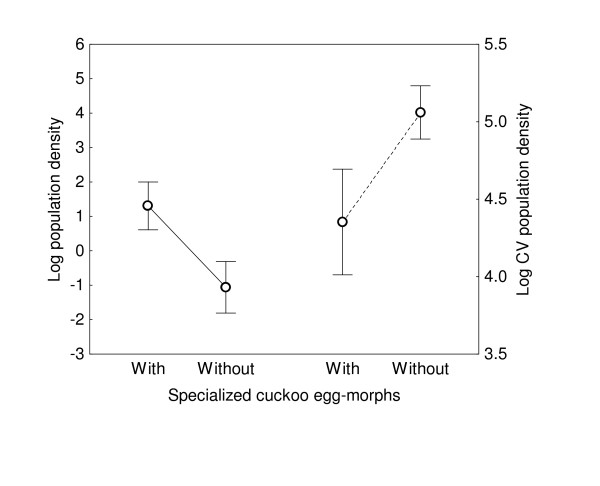
**Comparisons of host with and without cuckoo egg-morphs**. Mean and 95% confidence intervals for mean and coefficient of variation in population density of European passerine genera with species for which cuckoo-egg morphs have or have not been described.

When analyzing the relationship between the existence of cuckoo egg-morphs and population density and CV at the genus level, we found similar results. Populations of genera for which specialized cuckoo egg morphs had been described, either at the level of species or genus, were more dense and less heterogeneously distributed in Europe than populations of genera for which a cuckoo egg-morph had been described (Table 1; Fig. 2). Furthermore, the evolution of specialized cuckoo-egg morphs was more common in genera that held a large number of suitable host species (mean (SE): 4.70 (0.72) *vs *1.71 (0.29), t-test of log_10_-transformed data: t = 3.39, df = 29, P = 0.002), even after controlling for phylogenetic effects (Beta (SE) = 0.51 (0.16), t = 3.22, corrected df = 25, P = 0.004). The genera holding the largest number of species were also those with the most dense (average population density: log-transformed raw data: Beta (SE) = 0.43 (0.17), t = 2.72, df = 29, P = 0.017; using phylogenetic independent contrasts; Beta (SE) = 0.48 (0.16), t = 2.97, corrected df = 25, P = 0.01) and evenly distributed populations (lowest CV of population density: log-transformed raw data: Beta (SE) = -0.54 (0.16), t = 3.46, df = 29, P = 0.002; using phylogenetic independent contrasts; Beta (SE) = -0.71 (0.13), t = 5.40, corrected df = 25, P < 0.001).

## Discussion

We found evidence suggesting that cuckoo gentes have evolved more often in abundant suitable host species that are evenly distributed across the European continent. The interpretation of these results in a coevolutionary scenario of specialization largely depends on evidence supporting a genetic basis of cuckoo gentes on the one hand, and the appropriateness of our estimates of host density and heterogeneity in distribution on the other. Below we discuss these assumptions related to our estimates and the importance of our results for evolutionary scenarios of parasites specializing on suitable hosts.

Host density at the European continental range was estimated for twelve different regions, mostly including more than one country, while heterogeneity in distribution of suitable hosts was estimated as the coefficient of variation in population density in these twelve European regions. These regions were of similar size and comprised most of the European continent. The continental geographic scale used here has the advantage of producing general estimations largely independent of local variation at geographic local scales that do not capture general patterns in distribution of species. However, the level of heterogeneity in estimated population densities would most likely increase as the geographic scale decreases because heterogeneity in variables explaining species distribution (e.g. habitat requirements) also increases [17]. Therefore, if variation in habitat availability across geographic ranges is linked to variation in habitat selection of different host species, the geographic scale from which population density and level of heterogeneity in population density was estimated will be critical. A problem of non-stationary in our estimations would mainly affect estimates for patchily distributed species such as warblers associated with particular habitats (i.e. woodlands, wetlands) where they live at a high density. We explicitly attempted to quantify this potential problem in our estimations, and following recommendations by Osborne & Suarez-Seoane [17] we calculated density and coefficient of variation in density for more reduced geographic areas within the same European regions. We found that dividing regions into geographic sub-samples did not produce estimates that differed significantly from the original estimates (see Material and Methods). Therefore, we found no evidence of problems related to spatial non-stationary estimates in our estimates of population density and heterogeneity in distribution [17].

The formation of parasite races involves genetic changes that are part of adaptations to improve exploitation of a particular host or environment. Size and colour patterns of eggs have a strong genetic component [18,19] and vary greatly among host species [e.g., [20,21]]. Furthermore, strong evidence suggests that specific egg morphs with genes coding for egg colour patterns are maternally inherited and most likely located on the female-specific W-chromosome [22]. Thus, egg-morphs of cuckoos that mimic eggs of a single or few closely related host species provide evidence for such specialization and race formation in the European cuckoo [13]. Here we used the ten cuckoo egg morphs (i.e., gentes) assigned to a single host species by Moksnes & Røskaft [9] in a study of egg collection at museums. Because the very large sample size and broad geographic range used in that study, these cuckoo egg-morphs is considered as evidence of host race evolution by cuckoos across Europe [11].

Specialization could occur for hosts that the parasites will easily find [4]. Consequently, we predicted that cuckoo-egg morphs should have evolved for abundant host species [16]. In accordance, we found that cuckoo-egg morphs have more frequently evolved in species of higher average population density (see Results). These results were not due to the use of species-specific cuckoo-egg morphs exclusively because, when also including egg morphs associated with entire genera, population density of genera explained the probability of having specialized cuckoo egg morphs (Fig. 2).

Host density is a central species-specific trait explaining specialization and speciation of parasites because it affects probability of parasites locating preferred hosts. Specialization of parasites exploiting particular hosts invariably provokes disadvantages when parasitizing other hosts, and, consequently, the evolution of specialization can be seen as a compromise between the use of the maximum number of suitable hosts, and the use of the most frequent suitable host encountered [4]. Furthermore, for similar abundance of different suitable hosts, as the number of species increases, the probability of parasite specializing on a particular suitable host decreases. Consequently, relative host abundance is key to explaining host selection and host specificity of parasites [see examples in [3]]. Although host abundance or related variables, sometimes failed to explain host specificity [e.g., [23]], our results are consistent with the hypothesis.

Another demographic trait that could affect host specialization by parasites is the consistency in host abundance at different locations because, as density, it also affects the probability of parasites locating a host species and, therefore, the trade-off in the ability to optimally parasitize different hosts [3]. In agreement with this demographic trait being important for cuckoos, comparative evidence suggests that geographic range of potential hosts explains host selection by the European cuckoo in Britain [16]. Therefore, specialization and race (i.e., gentes) formation of cuckoos might have occurred in evenly distributed hosts (see Introduction). Again, our results were consistent with the prediction (Table 1, Fig. 2). These results were independent of the potential allometric effects of body size and nestling period for both host selection and demographic characteristics because the inclusion of these variables in our statistical model did not change the conclusions (see Results).

The hypothesis of specialization on the most predictable (i.e., abundant and evenly distributed) host resource was first introduced by Ward [5]. In a host-parasite system, large body size, long-lived or abundant hosts are considered the most predictable resources for parasites because living on a more predictable host may increase parasite fecundity and survival [23]. Because host adaptation might promote race formation and speciation by reducing gene flow [24,25], those evolutionary processes should more commonly occur in parasites that exploit the most predictable and abundant hosts [3]. In accordance with this line of reasoning, host specificity is more common in hosts that live longer (i.e., of large body mass) [23,26-29]. However, as far as we know, empirical evidence for the importance of heterogeneity in the distribution of hosts on parasite specialization does not exist in the literature, and, therefore, our results are the first to support this hypothetical relationship.

The scarcity of evidence supporting the importance of host density and distribution as variables related to predictability of resources (hosts) for parasites could be due to the parasite-host model systems being used in previous studies. Most of these studies were performed on internal parasites of fishes and flies [see, [23], and references therein] for which host body size is a variable that reflects predictability of resources for parasites. Evidence for the importance of host density explaining specialization by parasites came mainly from plant-phytophagous insect systems [see, [4]] where apart from size of hosts, host density *per se *is an important variable reflecting predictability of resources for adults or larvae. For cuckoos, abundance and distribution of suitable hosts are variables that closely reflect predictability of suitable host species (i.e., resources in brood parasite – host systems), which, as predicted by Norton and Carpenter [3], explains the association between these variables and probability of race formation in the European cuckoo. Thus, the association between patterns of geographic distribution of hosts (population density and heterogeneity) and evolutionary processes associated with specialization of parasites (i.e., speciation or evolution of races) should be predicted only in the case when population density and heterogeneity of distribution of hosts represent resource availability for parasites as in the case of brood parasites.

We have found an association between probability of specialized egg morph evolution and number of suitable host species within a genus. Although this result could suggest a possible role of cuckoos in the diversification of host genera, as in other parasite-host systems [2], it is more likely that the specialization of cuckoos on diversified host genera including abundant and homogeneously distributed species is of selective advantage. Closely related bird species tend to lay similar eggs [30]. Moreover, host ability to recognize foreign eggs depends on degree of mimicry between host and parasitic egg [e.g., [31,32]], and, thus, when egg-recognition ability improves in one but not in other host species within a given genus, cuckoo egg-morphs might still have success parasitizing some species in the genus.

## Conclusion

We have presented evidence suggesting that population density and homogeneity of geographic distribution of hosts explain the evolution of specialized egg-morphs of the European cuckoo. These results are consistent with the hypothesis that resource (i.e., host) predictability explains the evolution of host races and species in parasites.

## Methods

### Cuckoo egg morphs

Information on specialization by the European cuckoo in different host species is available from studies performed at different localities by different authors [33,34], but also from comparisons of cuckoo eggs stored at different museums that were collected from a very wide geographic range of localities [9]. We have in the present analyses followed Moksnes & Røskaft [9] who analyzed clutches from 27 different museum collections from 13 different European countries and established 15 cuckoo egg morphs; ten of these were assigned to a single species of host. The use of this classification has the advantage of being based on the work of the same observers with the possibility of comparing cuckoo eggs laid in nests of very different host species. Furthermore, this classification was made before we started our study, and thus the scientists performing the classification of cuckoo gentes were unaware of the hypothesis being tested here. After this classification some other species-specific cuckoo egg morphs have been proposed as gentes occurring at a very local scale, although these otherwise need further confirmation [33-35] and, therefore, were not considered in the present study.

Moksnes & Røskaft [9] also classified potential hosts as being unsuitable as cuckoo hosts, either because they are hole-nesters, or because they feed their young with food unsuitable for the cuckoo chick, or because they have nests/eggs that are too large to permit successful ejection by the young cuckoo [9]. Among suitable hosts, they distinguished between those with and without specific egg morphs. We considered all suitable hosts and the greenfinch *Carduelis chloris*, a granivorous species that is known to be able to successfully rear cuckoo nestlings [36]. Thus, our analyses comprise data for 79 passerine species (see Additional file 1). Moksnes & Røskaft [9] also described five different morphs of cuckoo eggs that were not species-specific, but associated with entire passerine genera (*Fringilla*, *Sylvia*, *Anthus*, *Lanius *and *Emberiza*). Thus, we considered in our analyses suitable host species for which cuckoos had and had not evolved species-specifics egg morphs. At the level of genera, we considered those with specialized described cuckoo-egg morphs together with others that included suitable species with specific cuckoo egg morphs as the genus for which cuckoos are specialized.

Information on body mass and the duration of the nesting period was included in the models because these variables are likely related to the quality of parental care (i.e., duration and feeding rates) that cuckoo nestlings receive when reared by different host species, and, therefore, they could affect host choice and the evolution of specialized cuckoo-egg morphs [16]. Data were obtained from Cramp [37] and Soler et al. [16].

### Demographic parameters

Estimation of population density and heterogeneity in density was based on maximum and minimum numbers of breeding pairs of different species reported by Tucker and Heath [38] and Hagemeijer and Blair [39]. We started with Tucker and Heath [38] by recording maximum and minimum numbers of breeding pairs for the 31 countries included in their appendix. Afterwards, for each species, we looked for information on countries not explicitly mentioned in Tucker and Heath's list, but within the top-ten list in Hagemeijer and Blair [39], where we recorded minimum and maximum population size to be included in our data set. For countries where the species was present [based on maps in [39]], but with no information on maximum and minimum numbers of breeding pairs in either of the two books, maximum and minimum values estimated for the group "other countries" in Hagemeijer and Blair [39] were distributed among countries proportionally to the area of these countries (Fig. 1). That was done after subtracting maximum and minimum population density of countries for which we collected data from Tucker and Heath (1994) that were not present in the top-ten list of Hagemeijer and Blair [39]. With these minimum and maximum population sizes we estimated geometric means for each species and country as (e^((log(minimum+1)+log(maximum+1))/2))^), which is appropriate for data of exponential nature as is the case for population size. Population density for each species and country was thus estimated as geometric means divided by area in square kilometres. For the estimation of demographic parameters at the level of genera, we pooled demographic data of potential hosts within the same genus. For each country we added minimum and maximum population sizes estimated for all species within the same genus. Population density for each genus and country was estimated as the geometric means of these maximum and minimum values divided by area in square kilometres.

We tried to reduce variation in estimates of population density related to size of different countries (i.e. the smallest countries might have reduced habitats diversity compared to large countries) by defining 12 different geographic regions of similar area as shown in Fig. 1. Importantly, by including more than one country in different geographic regions would allow us to estimate repeatability of estimations for the same regions (see below). For each of these geographic regions we summed population sizes (geometric means) as well as the area of each country within a region. Finally, for each species, we estimated means, standard deviations, and sums of population geometric means and population density. Afterward, we estimated the coefficient of variation as the percentage of the standard deviation divided by mean population density, which by definition reflects variation in population density of different areas (i.e., standard deviation) controlled for mean population density. Coefficient of variation (CV) is an index of spatial distribution widely used in ecology for characterizing uniformly (%CV < 100), randomly (%CV = 100) or contagiously (%CV > 100) distributed species [15]. Thus, low and high CV-values would indicate homogeneous and heterogeneous distributions, respectively.

Reliability of such heterogeneity values was tested by means of repeatability estimations. First, we estimated repeatability of values for different countries within the same regions. Briefly, for the nine European regions with more than one country we selected the two countries with the largest area. Then, we estimated the means and the coefficient of variation of densities of the nine regions for all analyzed species taking into account countries with the largest area within each region on the one hand, and countries with the second largest area within each European region on the other hand. Finally, repeatability of these values for species that appeared in both data sets was estimated by means of one-way ANOVAs. Both repeatability of mean (R = 51.0%, F = 3.38, df = 73, 74, P < 0.0001) and that of coefficient of variation of population density (R = 65.5%, F = 4.80, df = 73, 74, P < 0.0001) estimated for all analyzed species were highly significant. These results indicate that estimations for our twelve European regions are not influenced by the identity of countries within the same region or any associated difference (e.g., countries using different sampling methods or effort, or habitat diversity varying between countries) and validate the use of mean values per area.

Second, to rule out the possibility that our estimations depended on the European regions included in the analyses, we estimated means and coefficients of variation of population density for each species by taking into account regions with even and uneven identification numbers in Table 1, separately. Finally, we used one-way ANOVAs to estimate repeatability of means and coefficients of variation of population size estimated for different species using the two groups of European regions. Again, both mean (R = 84.6%, F = 12.01, df = 78, 79, P < 0.0001) and coefficient of variation of population density (R = 72.9%, F = 6.39, df = 78, 79, P < 0.0001) estimated for all species were significantly repeatable. These results indicate that our estimates of population density and heterogeneity do not depend on the European region for which data were collected and, consequently, our estimates can be considered species-specific characteristics.

### Comparative analyses

**Figure 3:**
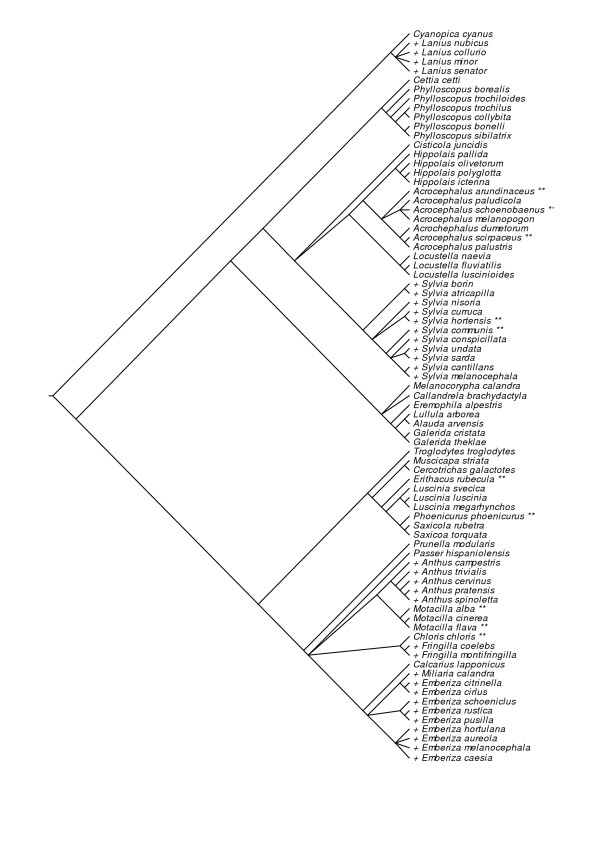
**Phylogenetic relationships between suitable hosts of the European cuckoo**. See Material and methods for sources. Species with species-specific cuckoo egg morphs are shown with two asterisks after the species names, while those with specialized cuckoo eggs for the entire genus are shown with + symbol before the species names.

Phylogenetic relationships among different species were based on recent publications [40-42] (Fig. 3). We assumed all polytomies (*N *= 14) to be unresolved. Branch lengths were assigned using three different methodologies: (i) all were set equal to one; (ii) by arbitrarily assigning all inter-node branch segments equal to one, but constraining tips to be contemporaneous [43]; and (iii) by tips being contemporaneous, the depth of each node being arbitrarily set to one less the number of tip species that descended from it [44].

To control for the possible effect of common phylogenetic descent, we used Felsenstein's [45] independent comparison method as implemented in the computer program PDAP (Vers. 6.0, module PDTREE) by Garland et al. [46] and Garland & Ives [47]. This method finds a set of independent pair-wise differences or contrasts, assuming that changes along the branches of the phylogeny can be modelled by a Brownian motion process (successive changes are independent of one another), and that the expected total change over many independent changes is zero [48]. Therefore, pair-wise differences in the phylogenetic tree are independent of each other [48]. The advantage of the independent comparison approach is that, by partitioning the variation appropriately, all contrasts can be used to assess a hypothetical comparative relationship [48]. These contrasts were estimated for each variable using the three kinds of trees differing in branch length (see above). Moreover, to check whether the contrasts were independent of branch length, we plotted absolute contrast values versus their standard deviations (square roots of sums of corrected branch lengths) [43,49,50]. After Bonferroni correction, in no case did we find a significant correlation when branch lengths were all adjusted to one, but when using arbitrary methods of Pagel and Grafen, absolute values of contrasts of population density were significantly related to the standard deviation (P < 0.05). Thus, for all variables we used estimated contrasts when branch lengths were arbitrarily assigned to one. These were subsequently used in multiple regression analyses through the origin, using the existence of specific cuckoo races for a target host species or genus (dependent variable: dummy continuous variable with values 1 and 0, respectively, indicating species with and without cuckoo gentes), and the mean and coefficient of variation in host population density (independent variables). Conservatively, we estimated degrees of freedom by subtracting the number of polytomies in the phylogenetic trees from those estimated by the statistical program, and we used two-tailed P-values.

After natural logarithmic transformations distributions of both mean and coefficient of variation of population density did not differ significantly from normality (Kolmogorov-Smirnov test for continuous variables, P > 0.15). Values reported are means (SE).

## Authors' contributions

JJS, APM, and MMV contributed equally to the conception and design of the study. JJS and MMV collected the necessary data from the European breeding bird atlas for estimates of population density of suitable hosts in different European regions. JJS wrote a first draft that was revised critically by APM and MMV. All authors have given final approval of the version to be published.

## Supplementary Material

Additional File 1 **Data used in the analyses**. Mean population density and coefficient of variation (CV) in density of breeding pairs of suitable hosts of the European cuckoo estimated for each of twelve European regions. Click here for file

## References

[B1] Brooks DR, McLennan DA (1993). Comparative study of adaptive radiations with an example using parasitic flatworms (Platyhelminthes: Cercomeria). Am Nat.

[B2] Brooks DR, McLennan DA (1993). Parascript Parasites and the language of evolution.

[B3] Norton DA, Carpenter MA (1998). Mistletoes as parasites: host specificity and speciation. Trends in Ecology & Evolution.

[B4] Jaenike J (1990). Host specialization in phytophagous insects. Ann Rev Ecol Syst.

[B5] Ward SA (1992). Assessing functional explanations of host specificity. Am Nat.

[B6] Thompson JN (1994). The coevolutionary process.

[B7] Tripet F, Christe P, Møller AP (2002). The importance of host spatial distribution for parasite specialization and speciation: a comparative study of bird fleas (Siphonaptera: Ceratophyllidae). J Anim Ecol.

[B8] Dawah HA, Hawkins BA, Claridge MF (1995). Structure of the parasitoid communities of grass-feeding chalcid wasps. J Anim Ecol.

[B9] Moksnes A, Røskaft E (1995). Egg-morphs and host preferences in the common cuckoo (Cuculus canorus: an analysis of cuckoo and host eggs from European museum collections. J Zool, Lond.

[B10] Marchetti K, Nakamura H, Gibbs HL (1998). Host-race formation in the common cuckoo. Science.

[B11] Davies NB (2000). Cuckoos cowbirds, and others cheats.

[B12] Moksnes A, Røskaft E, Rudolfsen G, Skjelseth S, Stokke G, Kleven O, Lisle Gibbs H, Honza M, Taborsky B, Teuschl Y, Vogl W, Taborsky M (2008). Individual female common cuckoos *Cuculus canorus *lay constant egg types but egg appearance cannot be used to assign eggs to females. J Avian Biol.

[B13] Brooke MdL, Davies NB (1988). Egg mimicry by cuckoos *Cuculus canorus *in relation to discrimination by hosts. Nature.

[B14] Whitlock MC (1996). The red queen beats the jack-of-all-trades: The limitations on the evolution of phenotypic plasticity and niche breadth. Am Nat.

[B15] Margalef R (1986). Ecologia.

[B16] Soler JJ, Møller AP, Soler M (1999). A comparative study of host selection in the European cuckoo *Cuculus canorus*. Oecologia.

[B17] Osborne PE, Suarez-Seoane S (2002). Should data be partitioned spatially before building large-scale distribution models?. Ecological Modelling.

[B18] Collias EC (1993). Inheritance of egg-color polymorphism in the village weaver (*Ploceus cucullatus*). Auk.

[B19] Gosler AG, Barnett PR, Reynolds SJ (2000). Inheritance and variation in eggshell patterning in the great tit Parus major. Proc R Soc Lond B.

[B20] Soler JJ, Moreno J, Avilés JM, Møller AP (2005). Blue and green egg-color intensity is associated with parental effort and mating system in passerines: Support for the sexual selection hypothesis. Evolution.

[B21] Kilner RM (2006). The evolution of egg colour and patterning in birds. Biol Rev.

[B22] Gibbs HL, Sorenson MD, Marchetti K, Brooke MdL, Davies NB, Nakamura H (2000). Genetic evidence for female host-specific races of the common cuckoo. Nature.

[B23] Simkova A, Verneau O, Gelnar M, Morand S (2006). Specificity and specialization of congeneric monogeneans parasitizing Cyprinid fish. Evolution.

[B24] Futuyma DJ, Moreno G (1988). The evolution of ecological specialization. Ann Rev Ecol Syst.

[B25] Poulin R (1998). Evolutionary ecology of parasites.

[B26] Desdevises Y, Morand S, Legendre P (2002). Evolution and determinants of host specificity in the genus Lamellodiscus (Monogenea). Biol J Lin Soc.

[B27] Simkova A, Desdevises Y, Gelnar M, Morand S (2001). Morphometric correlates of host specificity in Dactylogyrus species (Monogenea) parasites of European Cyprinid fish. Parasitology.

[B28] Krasnov BR, Morand S, Mouillot D, Shenbrot GI, Khokhlova IS, Poulin R (2006). Resource predictability and host specificity in fleas: the effect of host body mass. Parasitology.

[B29] Schneeweiss GM (2007). Correlated evolution of life history and host range in the nonphotosynthetic parasitic flowering plants Orobanche and Phelipanche (Orobanchaceae). J Evol Biol.

[B30] Harrison C (1975). A field guide to the nests, eggs, and nestlings of european birds with North Africa and the middle east.

[B31] Soler JJ, Martinez JG, Soler M, Møller AP (1999). Genetic and geographic variation in rejection behavior of cuckoo eggs by European magpie populations: An experimental test of rejecter-gene flow. Evolution.

[B32] Polacikova L, Honza M, Prochazka P, Topercer J, Stokke BG (2007). Colour characteristics of the blunt egg pole: cues for recognition of parasitic eggs as revealed by reflectance spectrophotometry. Anim Behav.

[B33] Alvarez F (1994). A gens of cuckoo *Cuculus canorus *parasitizing rufous Bush Chat *Cercotichas galactotes*. J Avian Biol.

[B34] Antonov A, Stokke BG, Moksnes A, Røskaft E (2006). Coevolutionary interactions between Common Cuckoos and Corn Buntings. Condor.

[B35] Alvarez F (1999). Attractive non-mimetic stimuli in Cuckoo Cuculus canorus eggs. Ibis.

[B36] Seel DC, Davis PRK (1981). Cuckoos reared by unusual hosts in Britain. Bird Study.

[B37] Cramp S (1998). Cramp's the complete birds of the Western Palearctic.

[B38] Tucker GM, Heath MF (1994). Birds in Europe, their conservation status.

[B39] Hagemeijer WJM, Blair MJ (1997). The EBCC atlas of European breeding birds: their distribution and abundance.

[B40] Alström P, Ericson PGP, Olsson U, Sundberg P (2006). Phylogeny and classification of the avian superfamily Sylvioidea. Molecular Phylogenetics and Evolution.

[B41] Jønsson KA, Fjeldså J (2006). A phylogenetic supertree of oscine passerine birds (Aves: Passeri). Zool Scripta.

[B42] Aliabadian M, Kaboli M, Prodon R, Nijman V, Vences M (2007). Phylogeny of Palaearctic wheatears (genus Oenanthe) – Congruence between morphometric and molecular data. Molecular Phylogenetics and Evolution.

[B43] Pagel M (1992). A method for the analysis of comparative data. J Theor Biol.

[B44] Grafen A (1989). The phylogenetic regression. Phil Trans R Soc Lond B.

[B45] Felsenstein J (1985). Phylogenies and the comparative methods. Am Nat.

[B46] Garland TJ, Midford PE, Ives AR (1999). An introduction to phylogenetically based statistical methods, with a new method for confidence intervals on ancestral states. Am Zool.

[B47] Garland TJ, Ives AR (2000). Using the past to predict the present: confidence intervals for regression equations in phylogenetic comparative methods. Am Nat.

[B48] Harvey PH, Pagel MD (1991). The comparative method in evolutionary biology.

[B49] Garland TJ, Huey RB, Bennett AF (1991). Phylogeny and coadaptation of thermal physiology in lizards: A reanalysis. Evolution.

[B50] Garland TJ (1992). Rate tests for phenotypic evolution using phylogenetically independent contrasts. Am Nat.

