# Supplementary material for: Geographic distribution of suitable hosts explains the evolution of specialized gentes in the European cuckoo Cuculus canorus
Source: BMC Evol Biol. 2009 Apr 30;9:88. doi: 10.1186/1471-2148-9-88 (PMC2683792; doi:10.1186/1471-2148-9-88)
Supplement: Additional File 1 — Data used in the analyses. Mean population density and coefficient of variation (CV) in density of breeding pairs of suitable hosts of the European cuckoo estimated for each of twelve European regions. [file 1471-2148-9-88-S1.doc]

Mean population density and coefficient of variation (CV) in density of breeding pairs of suitable hosts of the European cuckoo estimated for each of twelve European regions. Information on body mass (g, mean values of those reported for males and females in Perrins [1], and whether there are specific cuckoo host races (i.e. gentes) for each suitable cuckoo host according to Moksnes and Røskaft [2] is also provided

|  | Family | Density (pair /km^2^) | CV (density) | Specific gens | Nest. period | Body mass (g) |
| --- | --- | --- | --- | --- | --- | --- |
| *Acrocephalus arundinaceus* | Sylviidae | 0.20478 | 237.964 | yes | 12 | 31.0 |
| *Acrocephalus melanopogon* | Sylviidae | 0.00301 | 215.896 | no | 12 | 12.0 |
| *Acrocephalus paludicola* | Sylviidae | 0.00090 | 314.466 | no | 14 | 12.0 |
| *Acrocephalus palustris* | Sylviidae | 0.33954 | 138.324 | no | 12 | 13.0 |
| *Acrocephalus schoenobaenus* | Sylviidae | 0.60805 | 135.800 | yes | 13 | 12.0 |
| *Acrocephalus scirpaceus* | Sylviidae | 0.67182 | 236.267 | yes | 12 | 12.0 |
| *Acrocephalus dumetorum* | Sylviidae | 0.00271 | 236.602 | no | 11 | 12.0 |
| *Alauda arvensis* | Alaudidae | 9.04839 | 111.190 | no | 19 | 37.0 |
| *Anthus campestris* | Motacillidae | 0.09678 | 252.671 | no | 14 | 24.0 |
| *Anthus cervinus* | Motacillidae | 0.00311 | 259.261 | no | 13 | 20.0 |
| *Anthus pratensis* | Motacillidae | 1.90457 | 148.566 | no | 14 | 21.0 |
| *Anthus spinoletta* | Motacillidae | 0.15054 | 170.587 | no | 16 | 25.0 |
| *Anthus trivialis* | Motacillidae | 3.45273 | 119.544 | no | 13 | 23.0 |
| *Calcarius lapponicus* | Emberizidae | 0.09635 | 214.053 | no | 10 | 24.0 |
| *Calandrella brachydactyla* | Alaudidae | 0.43339 | 322.868 | no | 12 | 23.0 |
| *Cercotrichas galactotes* | Turdidae | 0.00337 | 304.729 | no | 12 | 24.0 |
| *Cettia cetti* | Sylviidae | 0.11365 | 196.431 | no | 15 | 15.0 |
| *Chloris chloris* | Fringillidae | 2.36584 | 78.124 | yes | 15 | 30.0 |
| *Cisticola juncidis* | Sylviidae | 0.50947 | 310.327 | no | 15 | 8.0 |
| *Cyanopica cyanus* | Corvidae | 0.04243 | 346.410 | no | 12 | 75.0 |
| *Emberiza aureola* | Emberizidae | 0.00004 | 337.121 | no | 13 | 23.0 |
| *Emberiza caesia* | Emberizidae | 0.00211 | 346.410 | no | 13 | 21.0 |
| *Emberiza cia* | Emberizidae | 0.25089 | 322.358 | no | 15 | 25.0 |
| *Emberiza cirlus* | Emberizidae | 0.28089 | 156.442 | no | 8 | 24.0 |
| *Emberiza citrinella* | Emberizidae | 3.61167 | 122.809 | no | 14 | 27.0 |
| *Emberiza hortulana* | Emberizidae | 0.12660 | 130.109 | no | 13 | 23.0 |
| *Emberiza melanocephala* | Emberizidae | 0.04775 | 342.029 | no | 12 | 28.0 |
| *Emberiza pusilla* | Emberizidae | 0.00177 | 340.214 | no | 14 | 14.0 |
| *Emberiza rustica* | Emberizidae | 0.06667 | 312.124 | no | 14 | 20.0 |
| *Emberiza schoeniclus* | Emberizidae | 0.73378 | 79.076 | no | 14 | 19.0 |
| *Eremophila alpestris* | Alaudidae | 0.00234 | 211.888 | no | 14 | 37.0 |
| *Erithacus rubecula* | Turdidae | 7.05374 | 63.316 | yes | 14 | 19.0 |
| *Fringilla coelebs* | Fringillidae | 17.0945 | 40.446 | no | 13 | 22.0 |
| *Fringilla montifringilla* | Fringillidae | 1.06949 | 208.747 | no | 12 | 26.0 |
| *Galerida cristata* | Alaudidae | 0.31759 | 152.566 | no | 12 | 40.0 |
| *Galerida theklae* | Alaudidae | 0.21874 | 346.402 | no | 13 | 37.0 |
| *Hippolais icterina* | Sylviidae | 0.30209 | 173.832 | no | 14 | 14.0 |
| *Hippolais olivetorum* | Sylviidae | 0.00238 | 346.410 | no | 19 | 18.0 |
| *Hippolais pallida* | Sylviidae | 0.03627 | 338.861 | no | 20 | 12.0 |
| *Hippolais polyglotta* | Sylviidae | 0.26513 | 261.410 | no | 15 | 13.0 |
| *Lanius collurio* | Laniidae | 0.58904 | 138.781 | no | 13 | 35.0 |
| *Lanius minor* | Laniidae | 0.01835 | 279.657 | no | 14 | 53.0 |
| *Lanius nubicus* | Laniidae | 0.00028 | 346.410 | no | 12 | 23.0 |
| *Lanius senator* | Laniidae | 0.10330 | 290.759 | no | 13 | 30.0 |
| *Locustella fluviatilis* | Sylviidae | 0.08192 | 197.705 | no | 14 | 19.0 |
| *Locustella luscinioides* | Sylviidae | 0.03930 | 281.916 | no | 10 | 15.0 |
| *Locustella naevia* | Sylviidae | 0.06644 | 157.238 | no | 13 | 13.0 |
| *Lullula arborea* | Alaudidae | 0.22570 | 260.949 | no | 15 | 30.0 |
| *Luscinia luscinia* | Turdidae | 0.13953 | 172.902 | no | 12 | 23.0 |
| *Luscinia megarhynchos* | Turdidae | 0.68389 | 121.489 | no | 13 | 23.0 |
| *Luscinia svecica* | Turdidae | 0.17816 | 202.389 | no | 14 | 19.0 |
| *Melanocorypha calandra* | Alaudidae | 0.28018 | 332.194 | no | 14 | 62.0 |
| *Miliaria calandra* | Emberizidae | 0.80689 | 182.974 | no | 15 | 47.0 |
| *Motacilla flava* | Motacillidae | 0.96897 | 117.877 | yes | 14 | 19.0 |
| *Motacilla alba* | Motacillidae | 1.88092 | 81.772 | yes | 12 | 23.0 |
| *Motacilla cinerea* | Motacillidae | 0.14590 | 145.037 | no | 13 | 19.0 |
| *Muscicapa striata* | Muscicapidae | 1.63674 | 131.829 | no | 14 | 16.0 |
| *Passer hispaniolensis* | Ploceidae | 0.39578 | 193.511 | no | 10 | 27.0 |
| *Phoenicurus phoenicurus* | Turdidae | 0.43849 | 65.444 | yes | 13 | 16.0 |
| *Phylloscopus bonelli* | Sylviidae | 0.32804 | 259.835 | no | 11 | 8.0 |
| *Phylloscopus borealis* | Sylviidae | 0.00096 | 342.877 | no | 13 | 11.0 |
| *Phylloscopus collybita* | Sylviidae | 3.38607 | 67.949 | no | 12 | 8.0 |
| *Phylloscopus sibilatrix* | Sylviidae | 1.37140 | 234.648 | no | 14 | 10.0 |
| *Phylloscopus trochiloides* | Sylviidae | 0.00621 | 301.503 | no | 12 | 8.0 |
| *Phylloscopus trochilus* | Sylviidae | 8.12539 | 116.365 | no | 14 | 8.0 |
| *Prunella modularis* | Prunellidae | 2.22413 | 111.184 | no | 17 | 17.0 |
| *Saxicoa torquata* | Turdidae | 0.25017 | 119.505 | no | 12 | 16.0 |
| *Saxicola rubetra* | Turdidae | 0.56481 | 138.712 | no | 12 | 20.0 |
| *Sylvia atricapilla* | Sylviidae | 4.20962 | 71.120 | no | 14 | 17.0 |
| *Sylvia borin* | Sylviidae | 2.10456 | 81.249 | no | 12 | 20.0 |
| *Sylvia cantillans* | Sylviidae | 0.30661 | 263.196 | no | 13 | 12.0 |
| *Sylvia communis* | Sylviidae | 1.44752 | 67.781 | yes | 12 | 15.0 |
| *Sylvia conspicillata* | Sylviidae | 0.03246 | 313.801 | no | 11 | 9.0 |
| *Sylvia curruca* | Sylviidae | 0.46179 | 79.490 | no | 19 | 13.0 |
| *Sylvia hortensis* | Sylviidae | 0.04383 | 304.406 | yes | 14 | 22.0 |
| *Sylvia melanocephala* | Sylviidae | 0.58954 | 174.303 | no | 13 | 11.0 |
| *Sylvia nisoria* | Sylviidae | 0.06315 | 232.399 | no | 14 | 28.0 |
| *Sylvia sarda* | Sylviidae | 0.00619 | 188.414 | no | 16 | 10.0 |
| *Sylvia undata* | Sylviidae | 0.37737 | 297.530 | no | 13 | 11.0 |
| *Troglodytes troglodytes* | Troglodytidae | 4.99850 | 174.320 | no | 10 | 11.0 |

Perrins CM (1987) Nueva generación de guías: Aves de España y de Europa. Omega, Barcelona

Reference List

1. Perrins CM: *Nueva generación de guías: Aves de España y de Europa*. Barcelona: Omega; 1987.

2. **On the evolution of blue cuckoo eggs in Europe**. In *J Avian Biol* 1995, **26:**13-19.
